# Benefits of Fruit and Vegetable Consumption on Prevalence of Metabolic Syndrome Are Independent of Physical Activity Behaviors in Older Adults

**DOI:** 10.3390/nu14020263

**Published:** 2022-01-09

**Authors:** Konstantinos-Georgios Papaioannou, Fawzi Kadi, Andreas Nilsson

**Affiliations:** School of Health Sciences, Örebro University, 701 82 Örebro, Sweden; konstantinos.papaioannou@oru.se (K.-G.P.); fawzi.kadi@oru.se (F.K.)

**Keywords:** dietary pattern, metabolic health, obesity, nutrition, sedentary time, aging, exercise, education level

## Abstract

Although consumption of fruits and vegetables (FV) is suggested to reduce metabolic risk, there is a paucity of studies taking advantage of objectively assessed physical activity (PA) behaviors when exploring links between FV intake and metabolic syndrome (MetS) in older adults. The aim of the present study was to determine the relationship between FV intake and MetS prevalence in a population of older community-dwelling adults, while considering time spent being sedentary and health-enhancing PA. Prevalence of MetS was determined in a population of 93 men and 152 women (age: 65–70 years). FV intake was determined by self-report and PA behaviors (time spent in moderate-to-vigorous PA (MVPA) and in sedentary) were assessed by accelerometry. Likelihood of having MetS by FV intake was determined using logistic regression with stepwise backward elimination including age, sex, educational level, total energy intake, adherence to MVPA guideline and total sedentary time as covariates. A main finding was that lower FV intakes were significantly related to higher prevalence of MetS (odds ratio [OR]: 1.23; 95% confidence interval [CI]: 1.03–1.47) after considering potential influences by covariates. Additionally, we found that lower intake of vegetables but not fruits was significantly related to higher prevalence of MetS (OR: 1.47; 95%CI: 1.04–2.07). In conclusion, lower intakes of FV in general, and of vegetables in particular, significantly increased likelihood of MetS, regardless of time spent sedentary and adherence to the MVPA guideline. From a public health perspective, our findings emphasize adequate intakes of FV as an independent contributor to metabolic health status in older adults.

## 1. Introduction

Metabolic syndrome (MetS) represents a cluster of established risk factors, characterized by abdominal obesity, elevated blood pressure, hyperglycemia, and disturbed blood lipid profile, predisposing development of cardiovascular and metabolic diseases [1]. Although MetS can be operationalized differently in term of defined variables and thresholds, it has been estimated that around 25% of the global adult population have MetS [2]. It has also been shown that the prevalence of MetS in the US population has increased over the past decades across all sociodemographic groups [3]. Importantly, several large-scale population studies have highlighted the increased prevalence of MetS by advancing age, regardless of the definition used [4,5,6,7]. In term of preventive strategies, dietary and physical activity behaviors are two key lifestyle factors currently promoted by major health organizations in order to combat the age-related increased risk of metabolic disorders. In this respect, it is globally recommended for adults to spend a minimum of 150 weekly minutes in moderate-to-vigorous physical activity (MVPA) alongside minimizing time spent being sedentary [8]. Moreover, common global guidelines about healthy eating, i.e., eating a diet rich in fruits and vegetables (FV), have been specifically emphasized for prevention of cardiometabolic disorders [9,10]. Indeed, diets with poor FV consumption are estimated to account for approximately half of all diet-related deaths globally [11] and have been associated with occurrence of type 2 diabetes mellitus (T2D) [12,13]. Interestingly, higher consumption of either fruits [14], vegetables [15] or both fruits and vegetables [16,17] have been associated with lower risk of MetS. These findings are of special interest for the older population as inadequate FV intakes have been reported in this age group [10]. However, despite the studies supporting that high FV intake reduces metabolic risk, there is currently no consensus regarding the relationship between fruit and vegetable intake and MetS and its components [15,17,18,19,20]. In particular, less evidence is available in aging populations and more importantly, the confounding effects by physical activity behaviors, encompassing time spent in different PA intensities, have often been overlooked. This is unfortunate given the established impact of PA on metabolic risk outcomes [21]. Importantly, there is mounting evidence of the detrimental impact of time spent sedentary on metabolic health regardless of time spent in MVPA [22]. Therefore, determining the role of FV on MetS prevalence should be strengthened by objective measures of daily time in PA and sedentary behaviors. To date, there is a paucity of studies taking advantage of objectively assessed PA behaviors when exploring links between FV intake and MetS.

The aim of the present study is to determine the relationship between FV intake and MetS prevalence in a population of older community-dwelling adults, while considering time spent being sedentary and in MVPA.

## 2. Materials and Methods

### 2.1. Participants

A total of 252 community-dwelling older adults (men and women) aged 65–70 years old were recruited through local advertisement. Participants were free from overt diseases such as diabetes mellitus or coronary heart disease and had no mobility disabilities. A written informed consent was provided by each participant and all research procedures were conducted in accordance with the principles set by the Declaration of Helsinki. The study was approved by the Swedish Ethical Review Authority (Dnr 2017/511).

### 2.2. Assessment of Dietary Intake

Number of FV servings per day was assessed based on the following two questions derived from the Swedish national survey on health and living conditions [23]: “How often do you eat fruit and berries, including all types of fruit and berries (fresh, frozen, preserved, juices, compote, etc.)?”; “How often do you eat vegetables and root vegetables, including all types of vegetables, legumes and root vegetables except potatoes (fresh, frozen, preserved, stewed, vegetable juices, vegetable soups, etc.)?” The following serving frequencies were used: less than one serving per day, 1 serving per day, 2 servings per day, 3 servings per day, 4 servings per day, 5 servings per day or more. In addition, total energy intake was assessed using a 90-item food-frequency questionnaire (FFQ), as previously described [24].

### 2.3. Assessment of Anthropometry and MetS

Height and weight were assessed using standard procedures. Components of MetS were assessed as follows: waist circumference (WC) was measured in the morning after an overnight fast to the nearest 0.1 cm at the midpoint between the iliac crest and lower costal margin using measuring tape. Systolic and diastolic blood pressures were assessed manually using a mercury sphygmomanometer after a 15 min rest. Fasting levels of glucose, triglycerides and HDL-cholesterol were determined on a Vitros-5.1 analyser platform using chemistry kits from Ortho-Clinical Diagnostics, Johnson & Johnson. Participants were classified with MetS based on the IDF criteria for metabolic syndrome [1]. In brief, participants with abdominal obesity (WC ≥ 80 cm for women and ≥94 cm for men) plus any two of the following risk factors (triglycerides, plasma glucose, and systolic or diastolic blood pressures above indicated thresholds and/or HDL-cholesterol below indicated sex-specific threshold or use of medication related to these abnormalities) were classified as having MetS.

### 2.4. Assessment of Physical Activity Behaviors

Adherence to the PA guidelines regarding 150 weekly min of MVPA was assessed using a waist-mounted Actigraph GT3x (Actigraph, Pensacola, FL, USA) accelerometer for a week, as previously described [21]. Accelerometer count cut-off points for total daily sedentary time (SED) (<100 counts per min) and MVPA (>2019 counts per minute) were used [25]. Participants accumulating an average of 22 min of MVPA per day (approximating 150 min per week) were classified as meeting the MVPA guideline.

### 2.5. Other Covariates

Information about education level (university/college, high-school, secondary school) and tobacco use (current, past, never) was retrieved by self-report.

### 2.6. Statistical Analysis

Data are presented as means ± SD, unless otherwise stated. Binary logistic regression was used to determine the impact of FV intake on the likelihood of having MetS. A stepwise backward elimination model was employed with a probability threshold for removal set to *p* > 0.1. Besides FV intake, a set of covariates including, age, sex, educational level, tobacco use, total energy intake, adherence to MVPA guideline and total sedentary time were entered in the first step. Thereafter, each variable fulfilling criteria for removal was eliminated in a stepwise manner. At the final step, only variables below removal threshold were retained. In addition, we analyzed the impact of FV intake expressed as two fixed categories (≤2 and ≥3 servings/day) on likelihood of having MetS. A priori power calculation (G*Power 3.1) was performed based on an expected overall MetS prevalence of at least 20% and an anticipated MetS prevalence of 30% due to low FV intake. Based on these assumptions and an alpha level set to 0.05, a sample size of 140 subjects would be required to obtain a statistical power of at least 80%. All statistical analyses were performed using SPSS Statistics, version 27.0 (Armonk, NY, USA: IBM Corp.).

## 3. Results

A population of 94 men (67 ± 1.5 yrs; 179 ± 7 cm; 81.5 ± 11.4 kg) and 152 women (67 ± 1.6 yrs; 164 ± 6 cm; and 64.4 ± 10 kg) had complete data on components of MetS (Table 1). In terms of educational level, 65% of the population reported university level, 26% high school level and 9% secondary school level. Total energy intake for the whole population corresponded to 1751± 565 kcal. On average, participants spent 502 ± 71 min in sedentary activities and a total of 79% of the population adhered to the weekly recommended amount of 150 min of MVPA. A total of 27% of the study population were classified with MetS (33% men and 23% women). A total of 23% of the study sample used either lipid-lowering or antihypertensive medication.

**Figure 1:**
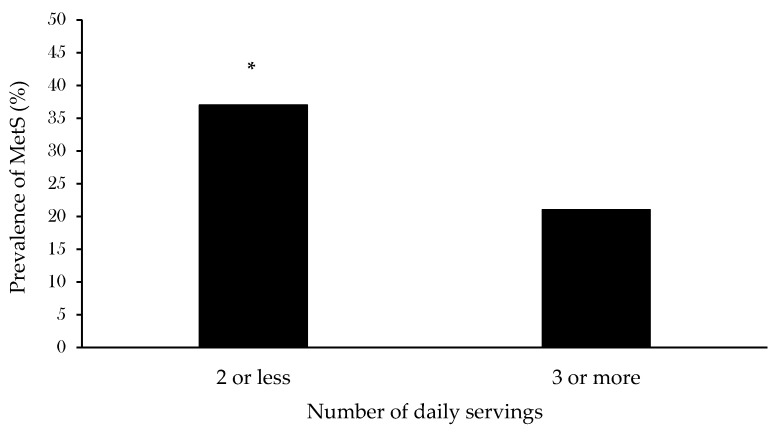
Prevalence of MetS in participants with lower (≤2) and higher (≥3) number of daily FV servings. * *p* < 0.05.

When exploring the impact of FV intake on the likelihood of having MetS, 10 participants were excluded from the logistic regression model due to incomplete data on covariates. Out of the eight variables entered in the first step, age, FV intake and adherence to the MVPA guideline were retained in the final model and significantly contributed to the likelihood of having MetS independently of each other. Importantly, the model revealed a significant inverse association between FV intake and MetS prevalence, with an increased likelihood of having MetS (odds ratio [OR]: 1.23; 95% confidence interval [CI]: 1.03–1.47) by lower FV intakes regardless of age and adherence to the MVPA guideline. On average, the number of FV servings/day in those with and without MetS were 2.6 ± 1.8 and 3.3 ± 1.8, respectively. We further compared MetS prevalence between fixed categories of FV intake (≤2 and ≥3 servings/day) (Figure 1). A significantly higher MetS prevalence was observed in those having a low FV intake compared to those with higher. Of note, 40% of the total population had an FV intake of ≤2 servings/day and the rest had higher intakes. In addition, we also compared MetS prevalence between those with a FV intake of 3–4 servings/day (41% of participants) and those with an intake of 5 servings/day or more (19% of participants) and did not observe significant differences in MetS prevalence between groups.

We further examined the impact of vegetable intake alone on the likelihood of having MetS. Vegetable intake, together with age and adherence to the MVPA guideline, was retained in the final model, where intakes of vegetables were inversely related to MetS prevalence (OR: 1.47; 95%CI: 1.04–2.07) independently of other covariates. On average, the number of daily vegetable servings in those with and without MetS was 1.2 ± 0.9 and 1.5 ± 0.9, respectively. However, no corresponding effects of fruit intake on MetS likelihood were observed. On average, the number of daily fruit servings in those with and without MetS was 1.4 ± 1.1 and 1.7 ± 1.2, respectively.

## 4. Discussion

The present work examined the influence of FV intake on the likelihood of having MetS when considering physical activity behaviors in community-dwelling older adults. A novel finding was that lower intakes of FV in general, and of vegetables in particular, significantly increased likelihood of MetS, regardless of time spent sedentary and adherence to the guideline of weekly MVPA.

Our findings support global guidelines emphasizing diets rich in FV for prevention of cardiometabolic disorders and are in accordance with data suggesting protective effects of increasing the number of daily FV servings on prevalence of MetS [14,15,16,17]. In particular, a daily intake of 5 FV servings corresponding to approximately 400g is widely recommended to promote health. In our population of older adults, around 20% had a FV intake of at least 5 servings/day, which is line with data from the Swedish food agency reporting that only 2 out of 10 Swedish adults adhere to the recommended amount [26]. Interestingly, our findings suggested a beneficial impact of a FV intake of at least 3 servings/day on MetS prevalence, while no further benefits were evident when increasing FV intake from 3–4 to 5 or more servings/day. Although much uncertainty still remains regarding the dose–response relationship between FV intake and different health outcomes, previous studies have shown beneficial FV-related health impacts with increased intakes up to 5 [27] to 8 servings/day [28], depending on the health outcome in question. Thus, data from our study population, comprising a substantial number of participants consuming ≤ 2 FV servings/day, suggest that increasing FV intake without necessarily reaching the recommended 5 servings/day will provide a beneficial impact on cardiometabolic health in older adults. Although exact mechanisms behind the beneficial effects of fruits and vegetables are yet to be determined, it is suggested that their multiple nutritional components, including vitamins and phytochemicals, can modulate oxidative stress [29,30], levels of systemic inflammation [31] and insulin resistance [32]. Moreover, fruits and vegetables are rich in dietary fibers, which have been related to reductions in cholesterol levels and blood pressure [33].

An interesting finding in our study was that the FV-related beneficial impact was driven by the consumption of vegetables rather than fruits. This result is in agreement with a recent longitudinal study reporting a higher risk of developing type 2 diabetes over a four-year period in men having a low intake of vegetables but not fruits [13]. Data on whether fruit or vegetable consumption is the main health-enhancing contributor have yielded contradictory results [14,16,18,34]. It may be hypothesized that varied types of fruits and vegetables, with a large variety of nutritional properties, have different health-enhancing potentials both alone and combined with other nutrients, which may partly explain the inconclusive data. Importantly, our finding that a higher intake of FV in general and vegetables in particular is associated with a reduced likelihood of having MetS is strengthened by the objective assessment of PA behaviors, which takes into account the importance of adherence to MVPA guideline for metabolic health [35]. Indeed, our analysis confirmed the significant and independent contributions of FV intake and adherence to MVPA on the likelihood of having MetS. Another novel finding is that the links between FV intake and MetS have been analyzed while considering the potentially detrimental inflammatory and metabolic effects of variations in time spent sedentary that may act independently of adherence to MVPA [22,36]. Altogether, our findings reinforce the importance of considering the dual independent roles of physical activity and dietary behaviors when tailoring health promotion efforts aiming to fight against development of metabolic disorders in older adults.

The results from the present study are further strengthened by the inclusion of potential confounding variables including age, sex, total energy intake, tobacco use and educational level. Notably, a previous study highlighted educational level as an important socio-economic factor able to readily moderate the link between FV intake and MetS [34]. Similarly, it has been shown that total energy intake may attenuate the relationship between FV intake and MetS [16]. However, due to its cross-sectional design, causality cannot be determined, and caution should be taken when generalizing our findings to broader populations of older adults. For example, 2/3 of the study sample reported university studies as the highest educational level, which is a proportion that is higher than that of the general population. However, only 20% of our participants adhered to the national guideline for FV servings (≥5 servings/day), which corresponds to population data on FV intake on national level [26]. Finally, residual confounding due to effects of dietary factors associated with healthy dietary patterns cannot be excluded.

## 5. Conclusions

In conclusion, the novel findings from the present study were that lower intakes of FV in general, and of vegetables in particular, significantly increased the likelihood of having MetS in community-dwelling older adults. Importantly, these detrimental effects were evident even after considering time spent sedentary and adherence to the MVPA guidelines. From a public health perspective, our findings emphasize adequate intakes of FV as an independent contributor to metabolic health status in older adults.

## Figures and Tables

**Table 1:** Components of the metabolic syndrome (MetS) in older men and women.

MetS Components	Men	Women
	(*n* = 94)	(*n* = 152)
Waist circumference (cm)	95 ± 10	80 ± 9
Systolic blood pressure (mmHg)	137 ± 11	136 ± 14
Diastolic blood pressure (mmHg)	84 ± 8	81 ± 9
Plasma glucose (mmol/L)	5.7 ± 0.6	5.3 ± 0.4
Triglycerides (mmol/L)	1.2 ± 0.5	1.0 ± 0.4
HDL-cholesterol (mmol/L)	1.5 ± 0.4	2.0 ± 0.4

## Data Availability

Data supporting reported results are available upon reasonable request and in accordance with the ethical principles.
